# Integrated Multi-Omics Investigation of Gypenosides’ Mechanisms in Lowering Hepatic Cholesterol

**DOI:** 10.3390/biom15081205

**Published:** 2025-08-21

**Authors:** Qin Jiang, Tao Yang, Hao Yang, Yi Chen, Yuan Xiong, Lin Qin, Qianru Zhang, Daopeng Tan, Xingdong Wu, Yongxia Zhao, Jian Xie, Yuqi He

**Affiliations:** Guizhou Engineering Research Center of Industrial Key-Technology for Dendrobium Nobile, Guizhou Engineering Research Center for Orchid Medicinal Plant Breeding and Efficient Application, Zunyi Medical University, Zunyi 563000, China; jiangqin@zmu.edu.cn (Q.J.); xiaoxinxin@zmu.edu.cn (T.Y.); haoyang@zmu.edu.cn (H.Y.); yichen@zmu.edu.cn (Y.C.); xiongyuan@zmu.edu.cn (Y.X.); qinlin@zmu.edu.cn (L.Q.); qrzhang_iris@foxmail.com (Q.Z.); tandp@zmu.edu.cn (D.T.); wuxingdong@zmu.edu.cn (X.W.); zhaoyongxia@zmu.edu.cn (Y.Z.)

**Keywords:** steroidogenesis pathway, gypenosides, cholesterol synthesis, transcriptomics, proteomics

## Abstract

(1) Objective: This study aimed to systematically elucidate the molecular mechanisms by which gypenosides (GP), a major active component of *Gynostemma pentaphyllum*, ameliorate hypercholesterolemia by modulating the hepatic steroidogenesis pathway, and to identify key therapeutic targets. (2) Methods: We established a high-fat diet (HFD)-induced hypercholesterolemia (HC) mouse model and performed GP intervention. An integrated multi-omics approach, combining transcriptomics and proteomics, was utilized to comprehensively analyze GP’s effects on the expression of genes and proteins associated with hepatic cholesterol synthesis, transport, and steroid hormone metabolism. (3) Results: HFD induced significant dysregulation, with 48 steroidogenesis pathway-related genes and 35 corresponding proteins exhibiting altered expression in HC mouse livers. GP treatment remarkably reversed these HFD-induced abnormalities, significantly restoring the expression levels of 42 genes and 14 proteins. Multi-omics integration identified seven critical genes/proteins—*Cyp3a25*, *Fdft1*, *Tm7sf2*, *Hmgcs1*, *Fdps*, *Mvd*, and *Pmvk*—that were consistently and significantly regulated by GP at both transcriptional and translational levels. Furthermore, correlation analyses demonstrated that *Cyp3a25* was significantly negatively correlated with serum total cholesterol (TC) and low-density lipoprotein cholesterol (LDL-C), whereas *Fdft1*, *Tm7sf2*, *Hmgcs1*, *Fdps*, *Mvd*, and *Pmvk* showed significant positive correlations. (4) Conclusions: GP effectively ameliorates cholesterol dyshomeostasis through a multi-targeted mechanism in the liver. It inhibits endogenous cholesterol synthesis by downregulating key enzymes (*Hmgcs1*, *Fdft1*, *Pmvk*, *Mvd*, *Fdps*, *Tm7sf2*), promotes cholesterol efflux and transport (upregulating *Abca1*, *ApoB*), and accelerates steroid hormone metabolism (upregulating *Cyp3a11*, *Cyp3a25*). These findings provide robust scientific evidence for the development of GP as a safe and effective novel therapeutic agent for hypercholesterolemia.

## 1. Introduction

Hypercholesterolemia has become a major global public health threat, with its prevalence closely linked to the widespread adoption of high-fat and high-sugar diets in modern life [1]. This condition not only escalates the risk of cardiovascular diseases but is also intimately associated with the onset of various chronic ailments, including metabolic syndrome and non-alcoholic fatty liver disease [2,3]. Current clinical practice predominantly relies on statins, which exert significant therapeutic effects by inhibiting 3-hydroxy-3-methylglutaryl coenzyme A reductase (HMGCR), a key enzyme in cholesterol synthesis [4]. However, long-term statin use can lead to adverse effects such as hepatotoxicity, muscle damage, and osteoporosis, thereby limiting its application in certain patient populations [5]. Consequently, exploring safe, effective, and less side-effect-prone natural alternatives for cholesterol reduction holds significant clinical importance.

*Gynostemma pentaphyllum* (Thunb.) Makino, a cucurbitaceous plant, is widely distributed in southwestern China, and its dried aerial parts are extensively used as a traditional Chinese medicine [6]. Compared to high-value traditional Chinese medicines like ginseng and *Panax notoginseng*, *G. pentaphyllum* offers advantages such as low cost, abundant availability, and strong environmental adaptability. Numerous studies have demonstrated that *G. pentaphyllum* and its primary active components, gypenosides, possess significant hypolipidemic, hypoglycemic, and hepatoprotective effects [7,8]. Notably, the basic structure of *G. pentaphyllum* total saponins is a dammarane-type tetracyclic triterpenoid saponin. *G. pentaphyllum* have been shown to reduce hepatic and serum cholesterol levels by promoting the conversion of cholesterol into bile acids. The research team previously isolated a novel dammarenyl-type triterpenoid compound from *G. pentaphyllum*, and it was found to inhibit the activity of key factors involved in regulating lipid metabolism [9,10,11].

**Figure 1:**
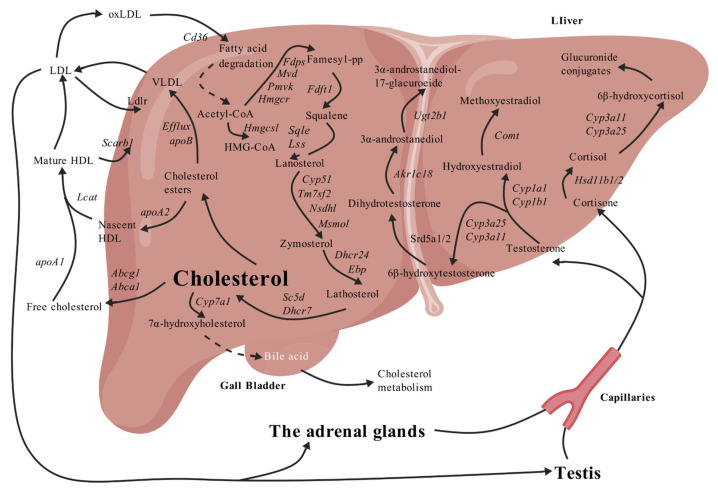
Steroidogenesis pathway of cholesterol synthesis and metabolism. Created with BioGDP.com (accessed on 15 July 2025) [29].

The liver serves as the central organ for cholesterol metabolism, responsible for its endogenous synthesis, exogenous uptake, transport, and catabolism [12]. Endogenous cholesterol synthesis primarily involves the conversion of acetyl-CoA to mevalonic acid, catalyzed by enzymes such as HMGCS1 and HMGCR, followed by a series of enzymatic reactions to form cholesterol [13,14]. The rate-limiting step of this process is catalyzed by HMGCR, making it a primary target for many cholesterol-lowering drugs [15]. A portion of the newly synthesized cholesterol is exported to extrahepatic tissues via the ABCA1 transporter, while another portion is esterified into cholesterol esters and assembled into high-density lipoproteins (HDLs) or low-density lipoproteins (LDLs). HDL facilitates the reverse transport of exogenous cholesterol back to the liver via the membrane receptor SR-B1, whereas LDL is taken up by the liver through the LDL receptor (LDLR) or utilized for steroid hormone synthesis in tissues like the testes and adrenal glands [16,17,18,19]. Furthermore, cholesterol can be metabolized into steroid hormones (e.g., testosterone and cortisol) by cytochrome P450 (CYP) family enzymes and hydroxysteroid dehydrogenases (HSDs). These hormones are subsequently further metabolized in the liver and excreted as glucuronide conjugates [20,21,22,23] (Figure 1). Therefore, the steroidogenesis pathway, encompassing cholesterol synthesis, transport, and metabolism, is crucial for investigating the regulation of cholesterol homeostasis [24,25,26].

The occurrence of hypercholesterolemia is intimately linked to abnormalities in the steroidogenesis pathway [27], while traditional Chinese medicines, with their multi-target and multi-level regulatory mechanisms, demonstrate unique therapeutic potential in restoring physiological functions [28]. Gypenosides may modulate cholesterol synthesis and transport by influencing gene expression and protein function within the steroidogenesis pathway, but their specific mechanisms warrant systematic investigation. To that end, this study aims to integrate clinical efficacy evaluation with animal experiments, combining transcriptomics and proteomics data. We will focus on the regulation of steroidogenesis pathway-related genes and proteins in the liver to analyze the effects of gypenosides on cholesterol synthesis, transport, and steroid hormone metabolism. This comprehensive approach will systematically elucidate the mechanisms by which gypenosides reduce hepatic cholesterol and uncover potential therapeutic targets, thereby providing a scientific basis for the development of novel lipid-lowering drugs.

## 2. Materials and Methods

### 2.1. Animals

Male C57BL/6J mice (4–8 weeks old, weighing 18–22 g), purchased from Beijing Huafu Kang Biological Technology Co., Ltd. (Beijing, China, License Number: SCXX (Beijing) 2014-0004), were acclimatized for one week in an SPF-grade animal facility (temperature 22 ± 2 °C, humidity 50 ± 10%, 12 h light/dark cycle). Subsequently, mice were randomly divided into four groups (*n* = 6): control (Con), high-cholesterol model (HC), gypenosides (GP), and simvastatin (SIM) as a positive control. The control group was fed a standard maintenance diet, while the remaining groups received high-fat diets with a fat content of 60%, protein content of 20%, and carbohydrate content of 20% for 16 weeks to induce hypercholesterolemia (purchased from Jiangsu Synergetic Biological Technology Co., Ltd., Nanjing, China). After successful modeling, interventions commenced: the control and HC groups were orally gavaged daily with 0.1% sodium carboxymethyl cellulose (CMC-Na) solution; the GP group received a daily oral gavage of gypenosides suspension (250 mg/kg, purity ≥ 98%, Purchased from Shanxi Zhongxin Biotechnology Co., Ltd., Taiyuan, China); and the SIM group received a daily oral gavage of simvastatin suspension (20 mg/kg, Sigma-Aldrich, St. Louis, MO, USA). The intervention lasted for 22 weeks, with body weight and food intake monitored weekly. At the end of the experiment, mice were fasted for 12 h before serum and liver samples were collected. Serum was used for biochemical indicator detection, and liver samples were stored at −80 °C for subsequent analyses. All animal experiments adhered to the International Animal Experiment Ethics Guidelines (ARRIVE guidelines) and were approved by the Institutional Animal Ethics Committee of Zunyi Medical University (Approval No. ZMU21-2203-610).

### 2.2. Transcriptomics Analysis

Total RNA was extracted from liver tissues (20 mg) of mice in each group. RNA concentration and purity (A260/A280 = 1.8–2.0) were assessed using a NanoDrop 2000 (Thermo Fisher, Waltham, MA, USA). NA integrity was verified by 1% agarose gel electrophoresis. Qualified RNA samples were sent to the Beijing Genomics Institution for transcriptomic sequencing using the BGISEQ-500 platform. Library preparation included mRNA enrichment, cDNA synthesis, end repair, adaptor ligation, and PCR amplification. Raw data were filtered for low-quality reads using fastp (v0.20.0), and clean reads were mapped to the mouse reference genome using HISAT2 (v2.1.0). Gene expression levels were quantified as FPKM (fragments per kilobase of transcript per million mapped reads) values. Steroidogenesis pathway-related genes were extracted from the Kyoto Encyclopedia of Genes and Genomes (KEGG) database. Differentially expressed genes (DEGs) were identified based on criteria of |log2FoldChange| > 2 and adjusted *p* value < 0.05 (DESeq2, v1.30.1). Volcano plots and heatmaps were generated using ggplot2, and principal component analysis (PCA) was performed using the prcomp function to evaluate differences in expression profiles between groups. Transcriptomic data were validated by quantitative real-time PCR (qRT-PCR) using the 2^−^^ΔΔ^*^Ct^* method, with *Gapdh* as the internal reference gene (Table 1). Three technical replicates were performed for each sample.

### 2.3. Proteomics Analysis

Liver tissues (50 mg) from each group were thawed from −80 °C, and 4 volumes of lysis buffer were added. Samples were then sonicated for lysis, followed by centrifugation at 12,000× *g* for 10 min at 4 °C. Supernatants were transferred to new tubes. Protein concentrations were determined using a BCA protein assay kit, and samples were stored at −80 °C. For digestion, 100 μg of protein was reduced with 5 mM dithiothreitol (DTT) for 30 min at 56 °C. Alkylation was performed by adding 11 mM iodoacetamide (IAA) and incubating in the dark at room temperature for 15 min. Urea was diluted to <2 M with 100 mM TEAB. Trypsin was added at a trypsin-to-protein ratio of 1:50 (*w*/*w*) and incubated overnight (approximately 16–18 h) at 37 °C. The next day, an additional amount of trypsin (1:100 *w*/*w*) was added, and digestion continued for 4 h at 37 °C.

The resulting peptide digests were re-suspended in mobile phase A (0.1% formic acid in water) and separated using an ultra-high-performance liquid chromatography (UPLC) system. A C18 reversed-phase column (75 μm × 15 cm, 3 μm particles) was used. Mobile phase B was 0.1% formic acid in acetonitrile. The gradient was set as follows: 0–70 min, 6–24% B; 70–84 min, 24–35% B; 84–87 min, 35–80% B; 87–90 min, 80% B. The flow rate was 450 nL/min. Separated peptides were introduced into a timsTOF Pro mass spectrometer via an electrospray ion source (voltage 1.6 kV). Data were acquired in parallel accumulation serial fragmentation (PASEF) mode, with a scan range of 100–1700 *m*/*z*. For each MS1 scan, 10 PASEF MS/MS spectra were acquired, targeting precursor ions with a charge state of 0–5. A dynamic exclusion window was set to 30 s. Mass spectrometry data were searched using MaxQuant (v1.6.6.0) against the SwissProt Mouse database (17,032 sequences), which included a reverse database and common contaminant proteins. Search parameters included trypsin/P as the enzyme with up to 2 missed cleavages; a first search precursor mass tolerance of 20 ppm; a fragment ion mass tolerance of 0.02 Da; and fixed modification with cysteine carbamidomethylation, while variable modifications were with methionine oxidation and protein N-terminal acetylation. False discovery rates (FDRs) for both proteins and PSMs were set at 1%.

### 2.4. Statistical Analysis

Gene and protein expression data were analyzed using R (v4.1.2). Clustering analysis was performed using “heatmap.2” (gplots 3.1.3 package), PCA using the “prcomp” function, and correlation analysis using the “cor” function (method = “pearson”). For comparisons between two groups, a two-tailed Student’s *t*-test was used. For comparisons among multiple groups, one-way analysis of variance (ANOVA) was performed using SPSS v18.0. A *p* value < 0.05 was considered statistically significant. Data are presented as mean ± standard error of the mean (SEM), with at least three independent experimental replicates.

## 3. Results

### 3.1. GP Reverses High-Fat Diet-Induced Dysregulation of Steroidogenic Pathway Gene Expression in Mouse Liver

**Figure 2:**
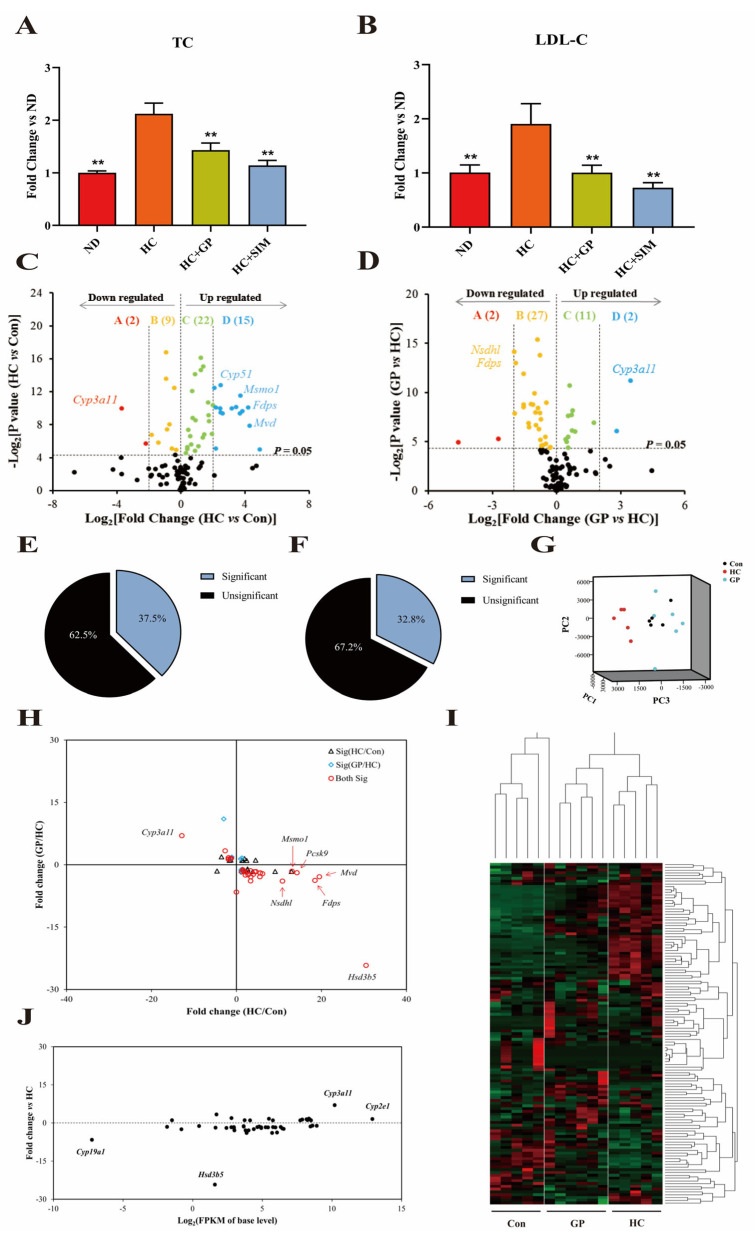
Transcriptomics analysis results. (**A**) The level of TC in serum. (**B**) The level of LDL-C in serum. **: *p* < 0.01 vs. HC. (**C**) Volcano plot of steroidogenesis pathway gene expression in HC vs. control group (*p* < 0.05). (**D**) Volcano plot of gene expression in GP vs. HC group (*p* < 0.05). (**E**) Proportion of gene expression changes in the HC group. (**F**) Proportion of gene expression changes in the GP group. (**G**) PCA score plot of gene expression. (**H**) Rank correlation analysis of differentially expressed genes. (**I**) Heatmap of gene expression. (**J**) Correlation analysis of GP regulatory effects on high-fat diet-modulated genes. In Figure 2I, red represents upregulated genes and green indicates downregulated genes. The gene expression pattern of the GP group was more closely aligned with that of the control group, suggesting that GP partially restored high-fat diet-induced transcriptional abnormalities.

After treatment with GP, compared with the HC group, the serum TC (Figure 2A) and LDL-C (Figure 2B) levels in the GP group were significantly decreased. Transcriptome sequencing identified the expression levels of 128 steroidogenesis pathway-related genes. After 38 weeks of continuous high-fat diet administration to normal mice, the expression levels of 48 steroidogenesis pathway genes in the livers of HC model mice were significantly altered compared to the control group (*p* < 0.05). This accounted for 37.5% of the genes in this pathway, with 37 genes upregulated (15 genes upregulated more than 4-fold) and 11 genes downregulated (2 genes downregulated more than 4-fold). Genes significantly affected by the high-fat diet included *Cyp3a11*, *Cyp51*, *Msmo1*, *Fdps*, and *Mvd* (Figure 2C,E).

Following GP treatment in HC mice, the expression levels of 42 steroidogenesis pathway genes in the livers of the GP group showed significant changes compared to HC mice (*p* < 0.05), accounting for 32.8% of the genes in this pathway. Among these, 13 genes were upregulated (2 genes upregulated more than 4-fold), and 29 genes were downregulated (2 genes downregulated more than 4-fold). Genes significantly affected by GP were *Cyp3a11*, *Fdps*, and *Nsdhl* (Figure 2D,F).

Spearman’s rank correlation analysis comparing differentially expressed genes (DEGs) between the HC/Con and GP/HC groups revealed that among the 48 genes significantly regulated by the high-fat diet, 31 genes had their expression levels significantly reversed or normalized after GP treatment (*p* < 0.05, Table 2). This indicates that GP can mitigate high-fat diet-induced transcriptional abnormalities (Figure 2H). The reversed genes primarily involved cholesterol synthesis-related genes such as *Hmgcs1* and *Fdft1*, and metabolism-related genes such as *Cyp3a11* and *Cyp7b1*.

Among the 48 genes significantly regulated by the high-fat diet, after GP treatment, the expression levels of *Cyp3a11* and *Cyp2e1* were significantly upregulated (log2 Fold Change > 10), while *Cyp19a1* was significantly downregulated (log2 Fold Change < −5), and *Hsd3b5* showed relatively small changes in expression, but there may still be some differences (Figure 2J). These genes might represent key targets for GP in modulating the steroidogenesis pathway.

Hierarchical clustering analysis (Figure 2I) demonstrated that the gene expression patterns of the control and HC groups formed two distinct clusters: genes highly expressed in the control group were concentrated in the lower quadrant of the heatmap, while those highly expressed in the HC group were concentrated in the upper quadrant. The gene expression pattern of the GP group was more closely aligned with that of the control group, forming two sub-clusters, suggesting that GP partially restored high-fat diet-induced transcriptional abnormalities.

PCA analysis (Figure 2G) showed that the gene expression patterns of the control, HC, and GP groups clustered into two distinct groups along the PC1 and PC2 axes. The GP group clustered closer to the control group, indicating that GP treatment significantly ameliorated the gene expression patterns within the steroidogenesis pathway, moving them towards a normal state.

### 3.2. RT-qPCR Validation Confirms Comparable Efficacy of GP and Simvastatin in Regulating Steroidogenic Gene Expression

**Figure 3:**
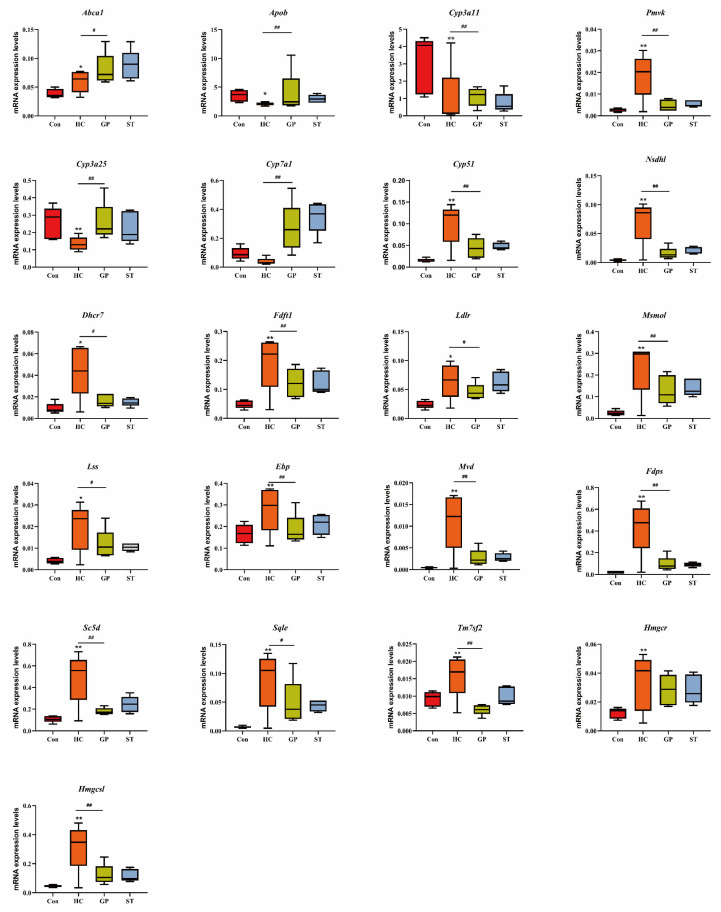
Expression levels of steroidogenesis pathway genes. Con: Control group. HC: Hypercholesterolemia model group. GP: Gypenosides treatment group. ST: Simvastatin treatment group. *: *p* < 0.05 vs. Con, **: *p* < 0.01 vs. Con; #: *p* < 0.05 vs. HC, ##: *p* < 0.01 vs. HC.

Based on the transcriptomic analysis results, 21 steroidogenesis pathway-related genes in the liver were significantly regulated by GP. Transcriptomic analysis revealed that, under continuous high-fat diet stimulation, all genes in the livers of HC model mice, except for *Abca1*, exhibited significant changes. Gypenosides significantly reversed the abnormal expression of high-fat diet-affected genes, with the exceptions of *Hmgcr* and *Abca1*. Specifically, GP significantly increased *Abca1* expression and showed an inhibitory effect on *Hmgcr* expression. RT-PCR analysis of mRNA expression levels in liver tissues from the normal control group (Con), hypercholesterolemia model group (HC), gypenosides treatment group (GP), and simvastatin positive control group (ST) demonstrated that the trends of mRNA expression changes in response to a high-fat diet and gypenosides were completely consistent with the transcriptomic analysis results. Furthermore, the regulatory effect of gypenosides on steroidogenesis pathway genes in the liver was comparable to that of the positive control drug, simvastatin (Figure 3).

### 3.3. Proteomics Analysis of Hepatic Steroidogenesis Pathway

**Figure 4:**
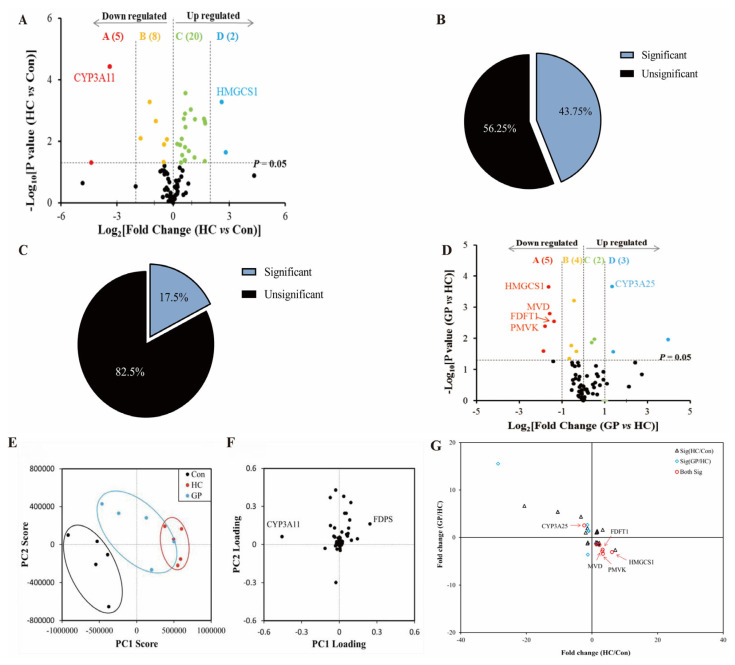
Proteomics analysis. (**A**) Volcano plot of steroidogenesis pathway protein expression in HC vs. control group (*p* < 0.05). (**B**) Proportion of protein expression changes in the HC group. (**C**) Proportion of protein expression changes in the GP group. (**D**) Volcano plot of protein expression in GP vs. HC group (*p* < 0.05). (**E**) PCA score plot of protein expression. (**F**) PCA loading plot of protein expression. (**G**) Rank correlation analysis of differentially expressed proteins.

LC-MS/MS analysis identified a total of 80 steroidogenesis pathway-related proteins. Compared to the control group, 35 proteins (43.75% of pathway proteins) in the livers of hypercholesterolemia model (HC) mice showed significantly altered expression (*p* < 0.05). Among these, 22 proteins were upregulated (2 proteins upregulated more than 4-fold), and 13 proteins were downregulated (5 proteins downregulated more than 4-fold). Proteins significantly affected by the high-fat diet included CYP3A11 and HMGCS1 (Figure 4A,B). Following gypenosides (GP) treatment, compared to the HC group, 14 proteins (17.5% of pathway proteins) in the livers of GP group mice exhibited significant expression changes, with 5 proteins upregulated (3 proteins upregulated more than 2-fold) and 9 proteins downregulated (5 proteins downregulated more than 2-fold). Proteins significantly affected by GP included CYP3A25, HMGCS1, MVD, FDFT1, and PMVK (Figure 4C,D).

Spearman’s rank correlation analysis comparing differentially expressed proteins (DEPs) between the HC/control and GP/HC groups revealed that among the 35 proteins significantly regulated by the high-fat diet, 13 proteins had their expression levels significantly reversed or normalized after GP treatment (*p* < 0.05, Table 3). The reversed proteins primarily involved cholesterol synthesis (e.g., FDFT1, HMGCS1) and metabolism (e.g., CYP3A25, CYP7A1), indicating that GP can mitigate high-fat diet-induced protein expression abnormalities (Figure 4G).

Principal component analysis (PCA) showed that the protein expression patterns of the control, HC, and GP groups clustered into three distinct groups along the PC1 axis, indicating significant inter-group differences (Figure 4E). The protein expression pattern of the GP group was more closely aligned with that of the control group, suggesting that GP treatment partially restored high-fat diet-induced protein expression abnormalities. The PCA loading plot (Figure 4F) further revealed that the expression level of CYP3A11 is positively correlated with lipid metabolism, and FDPS exhibits an opposing effect to CYP3A11.

### 3.4. Integrated Transcriptomic and Proteomic Analysis

**Figure 5:**
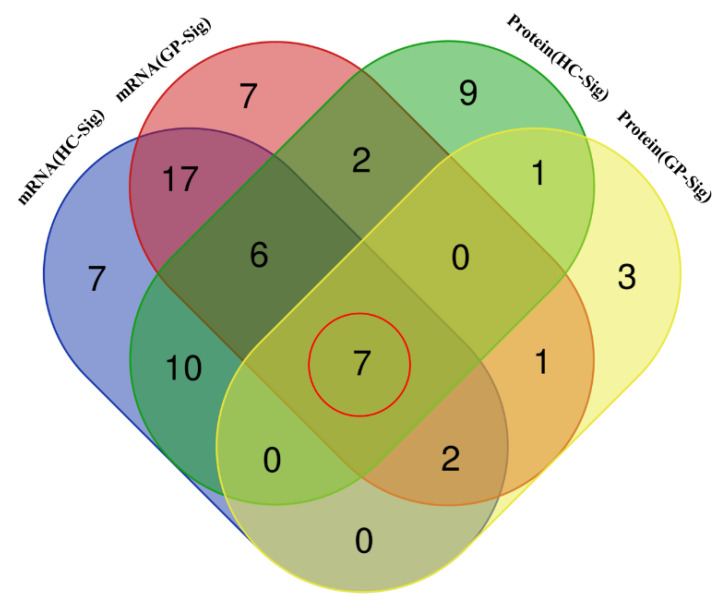
Venn diagram analysis of transcriptomics and proteomics. HC-Sig: Number of significantly changed steroidogenesis pathway genes/proteins in the HC group compared to the control group. GP-Sig: Number of significantly changed steroidogenesis pathway genes/proteins in the GP group compared to the HC group.

By integrating transcriptomic and proteomic data, we aimed to identify key targets of gypenosides in regulating the steroidogenesis pathway. Transcriptomic analysis showed that 48 steroidogenesis pathway genes in the livers of HC mice exhibited significant expression changes (*p* < 0.05), and 42 of these genes showed a trend of reversal after GP treatment, with 32 genes being significantly reversed (*p* < 0.05). Proteomic analysis revealed that 35 steroidogenesis pathway proteins in the livers of HC mice displayed significant expression changes, and 29 of these proteins showed a trend of reversal after GP treatment, with 8 proteins being significantly reversed (*p* < 0.05). Venn diagram analysis (Figure 5) identified 7 genes (*Cyp3a25*, *Fdft1*, *Tm7sf2*, *Hmgcs1*, *Fdps*, *Mvd*, *Pmvk*) that were significantly regulated by GP at both transcriptional and protein levels. This suggests that these genes/proteins represent potential targets for the cholesterol-lowering effects of GP.

**Figure 6:**
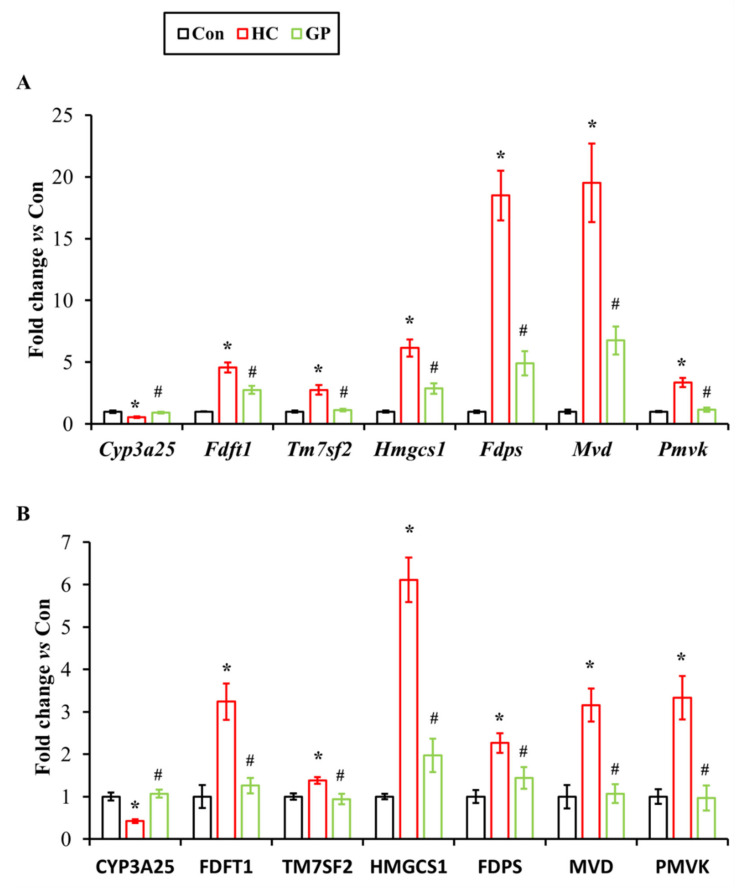
Expression changes in key target. (**A**) Changes in the expression of key target genes. (**B**) Changes in the expression of key target proteins. Con: Control group. HC: Hypercholesterolemia model group. GP: Gypenosides treatment group. *: *p* < 0.05 vs. Con. #: *p* < 0.05 vs. HC.

Cross-analysis of differentially expressed genes and proteins identified seven important genes significantly regulated by gypenosides. The changes in these genes and their encoded proteins induced by a high-fat diet were consistent, and their abnormal expression was significantly reversed after GP treatment. Compared to the control group, the transcript (Figure 6A) and protein (Figure 6B) expression of *Cyp3a25* were significantly decreased in the HC group, while GP treatment significantly upregulated them to near control group levels. Conversely, *Fdft1*, *Tm7sf2*, *Hmgcs1*, *Fdps*, *Mvd*, and *Pmvk* were significantly upregulated in the HC group and significantly downregulated after GP treatment.

**Figure 7:**
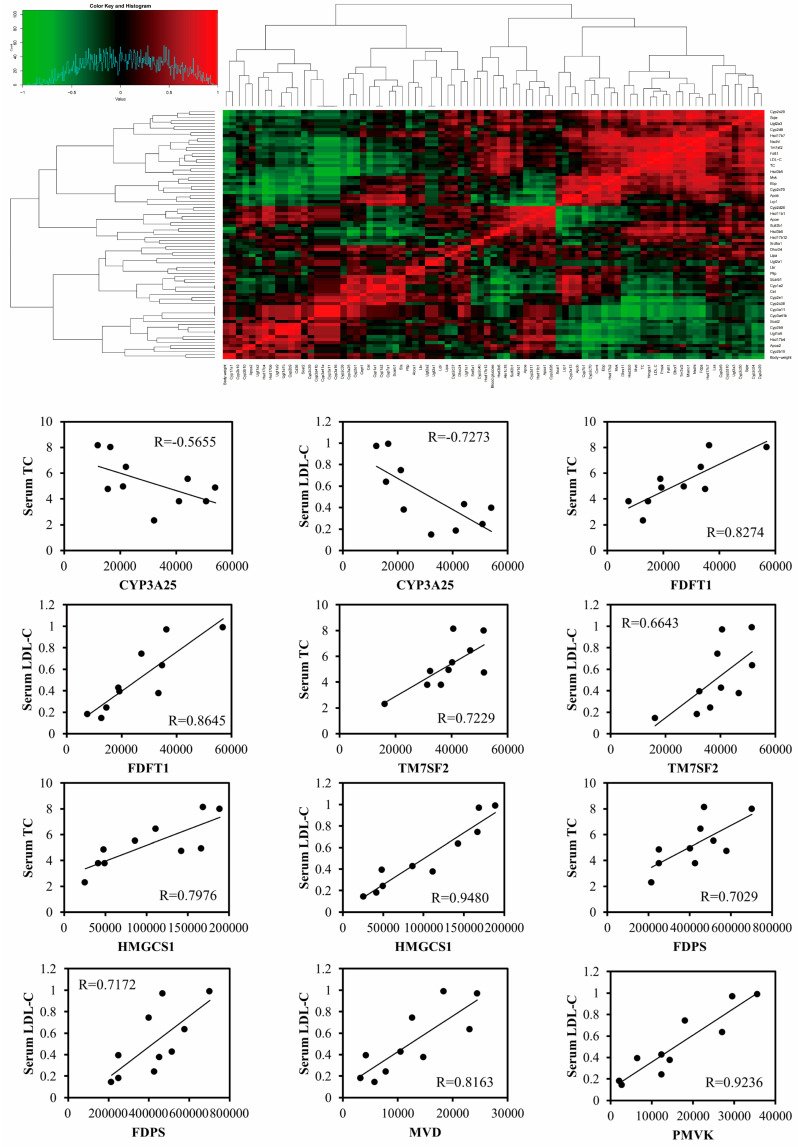
Correlation analysis between key target proteins and serum cholesterol. Red indicates positive correlation, green indicates negative correlation. *p* < 0.05.

Correlation analysis of hepatic steroidogenesis pathway proteins (Figure 7) revealed significant correlations among hepatic steroid biosynthesis pathway proteins, as well as between proteins and body weight, and between proteins and cholesterol levels. The heatmap showed that CYP3A25 was significantly negatively correlated with serum total cholesterol (TC) (*r* = −0.57, *p* = 0.02) and low-density lipoprotein cholesterol (LDL-C) (*r* = −0.73, *p* = 0.003). Conversely, FDFT1, TM7SF2, HMGCS1, FDPS, MVD, and PMVK exhibited significant positive correlations with TC and LDL-C (r range 0.66–0.95, *p* < 0.05). These results suggest that GP may reduce serum cholesterol levels by modulating these key targets.

## 4. Discussion

This study systematically elucidated the regulatory mechanisms of gypenosides (GP) on the hepatic steroidogenesis pathway in hypercholesterolemic (HC) mice by integrating transcriptomic and proteomic data. The regulation of cholesterol homeostasis by GP primarily manifested in three aspects: inhibition of endogenous cholesterol synthesis, promotion of cholesterol efflux and transport, and acceleration of steroid hormone metabolism. Previous research from our group has already confirmed that GP promotes cholesterol metabolism by modulating the bile acid pathway [8,10]. This study further clarified its comprehensive regulation of hepatic steroidogenesis pathway gene and protein expression through animal experiments. Transcriptomic analysis revealed that GP significantly modulated 42 steroidogenesis pathway genes (*p* < 0.05), among which 31 genes significantly reversed high-fat diet-induced abnormal expression. Proteomic analysis showed that GP significantly regulated 14 proteins (*p* < 0.05), with 8 proteins exhibiting significant reversal. These findings collectively indicate that GP restores the normal expression patterns of the steroidogenesis pathway through multi-target, multi-level regulatory mechanisms, thereby ameliorating high-fat diet-induced cholesterol metabolic disorders.

The mevalonate (MVA) pathway is the core pathway for endogenous cholesterol synthesis [30]. Our results demonstrated that under the influence of a high-fat diet, both the mRNA and protein expression levels of *Hmgcs1* were significantly elevated. This increase in HMGCS1 leads to a higher concentration of HMG-CoA, the substrate for the mevalonate reduction reaction, thereby accelerating cholesterol synthesis. Conversely, GP significantly inhibited the mRNA and protein expression levels of *Hmgcs1*. HMGCR, encoded by *Hmgcr*, is the rate-limiting enzyme of the MVA pathway, and statins significantly reduce cholesterol levels by inhibiting its activity [31]. Under high-fat diet stimulation, the mRNA expression level of *Hmgcr* was significantly increased. As HMGCR is the rate-limiting enzyme in the cholesterol synthesis pathway, this elevated mRNA expression likely accelerates the mevalonate reduction reaction, consequently speeding up cholesterol synthesis. First-line clinical cholesterol-lowering statins primarily function as inhibitors of this enzyme to exert their hypocholesterolemic effects [32,33]. Interestingly, our study found that *Hmgcr* mRNA expression levels tended to decrease in HC mice after treatment with both gypenosides and simvastatin. Following mevalonate formation, 5-pyrophosphomevalonate is produced through the catalysis of PMVK, subsequently converted to isopentenyl pyrophosphate by MVD. This is then catalyzed by FDPS to form farnesyl pyrophosphate, a crucial branching point in cholesterol synthesis [34]. Our study observed that under high-fat diet stimulation, the mRNA and protein expression levels of *Pmvk*, *Mvd*, and *Fdps* were all significantly upregulated, potentially accelerating farnesyl pyrophosphate formation. However, in HC mice treated with gypenosides, the mRNA and protein expression levels of all three genes (*Pmvk*, *Mvd*, *Fdps*) were significantly reduced. It is noteworthy that GP’s mechanism of inhibiting FDPS to lower cholesterol is consistent with that reported for alendronate [35].

After farnesyl pyrophosphate formation, squalene is produced through the catalysis of FDFT1. Our results align with many previous studies [36], showing that under high-fat diet stimulation, both the mRNA and protein expression levels of *Fdft1* were significantly elevated. Gypenosides significantly inhibited both the mRNA and protein expression levels of this gene. It is worth mentioning that clinical statins have been shown to inhibit cholesterol synthesis by suppressing FDFT1 protein expression [35]. Furthermore, during the conversion of squalene to cholesterol, gypenosides significantly mitigated the high-fat diet-induced abnormal upregulation of mRNA expression for *Lss*, *Cyp51*, *Tm7sf2*, *Nsdhl*, *Msmo1*, *Ebp*, *Sc5d*, and *Dhcr7* at the transcriptional level. Among these, both the mRNA and protein expression of *Tm7sf2* were significantly upregulated by the high-fat diet. This gene is considered a candidate gene for studying the relationship between high-energy diets and liver function and lipid profiles [37,38], and its downregulation further confirms GP’s inhibitory effect on cholesterol synthesis. In fact, hepatic cholesterol accumulation in HC mice was significantly improved after GP treatment [39]. These findings indicate that GP significantly reduces hepatic cholesterol synthesis by inhibiting the MVA pathway through multiple targets.

Cholesterol efflux and transport are critical processes for maintaining hepatic cholesterol homeostasis. The transporter ABCA1 initiates HDL assembly by promoting free cholesterol efflux to pre-β HDL. This study found that a high-fat diet inhibited *Abca1* mRNA expression, leading to impaired HDL synthesis, which is consistent with reported mechanisms of hepatic cholesterol accumulation [40,41]. GP treatment significantly upregulated *Abca1* mRNA expression, potentially accelerating HDL formation and promoting cholesterol transport from the liver to plasma, ultimately reducing TC and LDL-C levels. Additionally, *ApoB* is a core component for VLDL assembly, responsible for transporting cholesterol esters from the liver into the bloodstream [42]. Our study showed that a high-fat diet downregulated *ApoB* mRNA expression, potentially contributing to intrahepatic cholesterol accumulation, whereas GP significantly upregulated *ApoB* expression, promoting VLDL-mediated cholesterol efflux. Notably, the regulatory effects of GP on *Abca1* and *ApoB* were comparable to those of simvastatin, suggesting that GP may possess similar transport regulatory potential to clinical lipid-lowering drugs.

LDL plays a crucial role in blood cholesterol transport, primarily responsible for delivering cholesterol to various organs to meet the body’s needs while preventing its accumulation in the blood and liver [43]. Following a high-fat diet, serum LDL-C levels in mice significantly increased. This could be attributed to the significant inhibition of steroid hormone metabolism-related genes such as *Cyp3a11* and *Cyp3a25* by the high-fat diet [44], leading to impaired steroid hormone metabolism. The resulting accumulation of steroid hormones may reduce the demand for cholesterol by the testes and adrenal glands. Research indicates that approximately 80% of steroid hormones synthesized in the human body derive from LDL-provided cholesterol [45]. Therefore, the suppressed expression of *Cyp3a11* and *Cyp3a25* might contribute to elevated blood LDL-C levels. LDL is also known as “bad” cholesterol because it carries a large amount of cholesterol (accounting for approximately 50% of its volume) and primarily transports cholesterol to various organs via the bloodstream. Consequently, elevated LDL can lead to excessive cholesterol in the blood, potentially causing vascular blockage and increasing the risk of peripheral vascular diseases like atherosclerosis [46]. This study demonstrated that in HC mice treated with gypenosides, the mRNA expression level of *Cyp3a11* significantly increased, and both the mRNA and protein expression levels of *Cyp3a25* significantly increased. These effects likely contribute to the significant reduction in serum LDL-C levels observed in mice.

**Figure 8:**
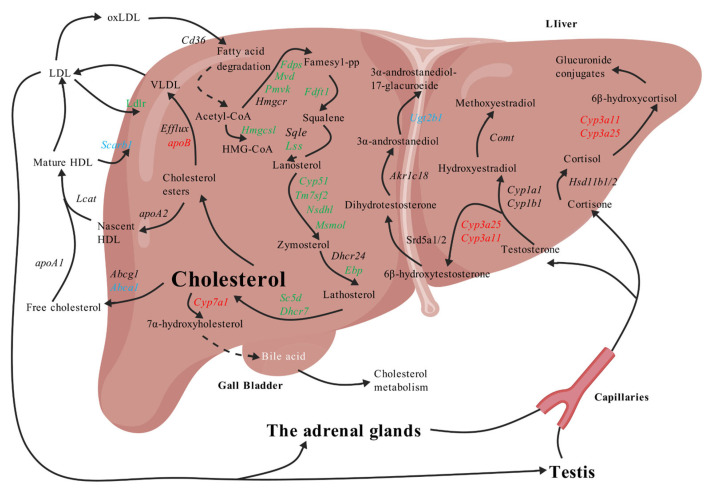
Regulatory mechanisms of gypenosides on steroidogenesis pathway, created with BioGDP.com [29]. Red indicates genes/proteins downregulated by high-fat diet and upregulated by GP. Green indicates genes/proteins upregulated by high-fat diet and downregulated by GP. Blue indicates genes/proteins not significantly affected by high-fat diet but upregulated by GP.

The regulatory mechanisms of GP on the steroidogenesis pathway share similarities with clinical lipid-lowering drugs in inhibiting cholesterol synthesis [30,35]. Simultaneously, its multi-target action on the synthesis, transport, and metabolism of steroids highlights the unique advantages of traditional Chinese medicine. However, despite revealing GP’s lipid-lowering mechanisms through multi-omics analysis, this study has limitations. Firstly, this study was based on a mouse model, and further clinical trials are needed to validate the efficacy and safety of GP in humans. Additionally, the functional validation of key targets such as *Cyp3a25* and *Hmgcs1* could be further explored through gene knockout or CRISPR/Cas9 technology to clarify their precise roles in GP’s lipid-lowering mechanism. Finally, integrating metabolomics and lipidomics data could further comprehensively reveal GP’s regulation of the cholesterol metabolic network (Figure 8).

## 5. Conclusions

In this study, through a robust integration of transcriptomic and proteomic analyses, we systematically elucidated the intricate molecular mechanisms by which gypenosides (GP) effectively alleviate hepatic cholesterol accumulation. Our findings demonstrate that GP exerts its hypocholesterolemic effects via a multi-targeted approach, significantly ameliorating high-fat diet-induced dysregulation of the steroidogenesis pathway. Specifically, GP robustly suppresses endogenous cholesterol synthesis by downregulating key enzymes such as HMGCS1, FDFT1, PMVK, MVD, FDPS, and TM7SF2. Concurrently, it promotes cholesterol efflux and transport through the upregulation of ABCA1 and ApoB, and it accelerates steroid hormone metabolism by increasing the expression of CYP3A11 and CYP3A25. Crucially, our multi-omics data converged on seven key targets—Cyp3a25, Fdft1, Tm7sf2, Hmgcs1, Fdps, Mvd, and Pmvk—which were consistently and significantly modulated by GP at both transcriptional and protein levels, and strongly correlated with reduced serum total cholesterol (TC) and low-density lipoprotein cholesterol (LDL-C). These comprehensive insights provide a strong scientific foundation for the development of GP as a promising, safe, and effective novel therapeutic agent for hypercholesterolemia.

## Figures and Tables

**Table 1:** Primers for qPCR.

Gene Name	Forward Primer Sequence (5′ to 3′)	Reverse Primer Sequence (5′ to 3′)
*Gapdh*	TGTGTCCGTCGTGGATCTGA	CCTGCTTCACCACCTTCTTGA
*Cyp7a1*	GAGCCCTGAAGCAATGAAAG	GCTGTCCGGATATTCAAGGA
*Cyp3a11*	TGCCTTGGCATGAGGTTTGC	TGACTGCATCCCGTGGCACA
*Fdps*	CCGGGAGAATCCGCGTTGAA	CCTTGAGCCGGGCAATAGCA
*ApoB*	GCTCAACTCAGGTTACCGTGA	AGGGTGTACTGGCAAGTTTGG
*Cyp3a25*	TCGGGGGCTATGATGCCACA	CCATGTCCATCAGGGCGTCA
*Msmo1*	TGGCAAGGTGTTTGGGCTGTGC	ATGGTCACCCATGCCCACAGGA
*Sc5d*	TCAGCATCCCCACCGTCTCA	CGGCGTGGGGATCTTCCAAA
*Ebp*	ACTGGCCTTGTGCTGGTTT	TCCATACAGACGACGAAGCTG
*Tm7sf2*	CCATTGTTCCCCGGCAAAGC	TGCAGGCCGAGCCAGATGAA
*Hmgcs1*	TCCGTGCCCAGTGGCAGAAA	CCCCAAAGGCTTCCAGTCCA
*Dhcr7*	TCCCAACGGCAAGGCTGGAT	ATCCAATGCGGGGGCAGTCA
*Nsdhl*	TGGCATTTTCGGCCCAAGG	ATGCGGGACAGGAACGTCCA
*Sqle*	AGTTCGCTGCCTTCTCGGATA	GCTCCTGTTAATGTCGTTTCTGA
*Cyp51*	AACGAAGACCTGAATGCAGAAG	GTGGGCTATGTTAAGGCCACT
*Fdft1*	TGTGGCCGTGCAGTGCTTGA	TGACGGCAGGCATGTTGGTG
*Ldlr*	TTGGGGAACACCCGCCAAGA	TCCGATTGCCCCCATTGACA
*Pmvk*	CAGCTTTAGGCCTGGTGAAG	CCTTGAGTGGACCAGAGAGC
*Hmgcr*	CGGCAGCTTGCCCGAATTGT	AACCCAATGCCCGCGCTTCA
*Mvd*	ACCCGCTGCATCCAAGAGCA	GCGTTGGGGCCAGCATCAAA
*Lss*	TGCTGGGCCCTGATGGGTTT	AAATGTGGCCGGCGAGGGTA
*Abca1*	CGGCATCAATGGCAGTGTGG	CCACGGCCATGGCAAAAAGG

**Table 2:** The steroid pathway genes significantly regulated by gypenosides.

Number	Gene ID	Gene Symbol	Fold Change		*p*-Value	
			HC/Con	GP/HC	HC/Con	GP/HC
1	13106	*Cyp2e1*	-1.23	1.54	3.31 × 10^−2^	6.01 × 10^−4^
2	13112	*Cyp3a11*	-12.87	7.01	1.01 × 10^−3^	1.48 × 10^−2^
3	226105	*Cyp2c70*	3.35	-3.89	1.79 × 10^−3^	4.32 × 10^−3^
4	110196	*Fdps*	18.49	-3.77	9.29 × 10^−4^	1.21 × 10^−4^
5	238055	*ApoB*	-1.45	1.47	2.94 × 10^−2^	5.11 × 10^−3^
6	56388	*Cyp3a25*	-1.83	1.70	5.95 × 10^−3^	3.50 × 10^−3^
7	66234	*Msmo1*	13.07	-1.55	3.46 × 10^−4^	4.07 × 10^−2^
8	235293	*Sc5d*	4.00	-2.29	7.77 × 10^−4^	2.24 × 10^−3^
9	13123	*Cyp7b1*	1.86	-1.62	3.51 × 10^−2^	4.13 × 10^−2^
10	13595	*Ebp*	1.84	-1.70	5.54 × 10^−5^	7.22 × 10^−5^
11	71773	*Ugt2b1*	1.25	-1.71	4.33 × 10^−2^	4.20 × 10^−3^
12	73166	*Tm7sf2*	2.76	-2.44	8.81 × 10^−3^	1.14 × 10^−2^
13	208715	*Hmgcs1*	6.15	-2.14	1.53 × 10^−3^	2.32 × 10^−3^
14	15488	*Hsd17b4*	-1.90	1.34	8.97 × 10^−6^	2.72 × 10^−2^
15	13360	*Dhcr7*	5.51	-2.90	1.01 × 10^−3^	2.32 × 10^−3^
16	18194	*Nsdhl*	10.84	-3.92	9.04 × 10^−4^	5.60 × 10^−5^
17	13121	*Cyp51*	5.60	-2.02	1.43 × 10^−4^	1.06 × 10^−3^
18	14137	*Fdft1*	4.58	-1.66	9.32 × 10^−4^	6.44 × 10^−3^
19	16835	*Ldlr*	3.28	-1.41	6.04 × 10^−4^	3.70 × 10^−2^
20	15496	*Hsd3b5*	30.49	-24.21	3.22 × 10^−2^	3.29 × 10^−2^
21	68603	*Pmvk*	3.37	-2.91	2.98 × 10^−3^	2.61 × 10^−4^
22	243085	*Ugt2b35*	-1.90	1.67	8.32 × 10^−5^	1.43 × 10^−2^
23	100102	*Pcsk9*	14.26	-1.94	1.24 × 10^−3^	9.86 × 10^−3^
24	192970	*Dhrs11*	1.64	-1.39	2.37 × 10^−4^	2.02 × 10^−3^
25	192156	*Mvd*	19.51	-2.89	4.27 × 10^−3^	2.78 × 10^−3^
26	16987	*Lss*	5.60	-1.96	1.40 × 10^−3^	3.99 × 10^−3^
27	244209	*Cyp2r1*	2.39	-1.84	1.42 × 10^−5^	2.33 × 10^−5^
28	15493	*Hsd3b2*	4.31	-1.69	1.77 × 10^−4^	2.79 × 10^−2^
29	98386	*Lbr*	1.49	-1.22	1.05 × 10^−2^	4.68 × 10^−2^
30	72082	*Cyp2c55*	2.13	-2.49	2.41 × 10^−2^	8.48 × 10^−3^
31	13098	*Cyp2c39*	-2.66	3.35	1.76 × 10^−2^	8.29 × 10^−3^

Note: Control group (Con). Hypercholesterolemia model group (HC). Gypenosides treatment group (GP). “-” indicates downregulation.

**Table 3:** The steroid pathway protein significantly improved by gypenosides.

Number	Protein Accession	Gene Symbol	Fold Change		*p*-Value	
			HC/Con	GP/HC	HC/Con	GP/HC
1	Q71KT5	*TM7SF2*	1.38	-1.47	8.32 × 10^−3^	1.71 × 10^−2^
2	Q05421	*CYP2E1*	-1.00	1.31	9.96 × 10^−1^	1.36 × 10^−2^
3	Q61009	*SCARB1*	-1.05	1.44	7.28 × 10^−1^	1.06 × 10^−2^
4	P53798	*FDFT1*	3.24	-2.57	2.22 × 10^−3^	2.84 × 10^−3^
5	Q61694	*HSD3B5*	-1.27	-3.62	3.31 × 10^−1^	2.57 × 10^−2^
6	Q64505	*CYP7A1*	-1.38	2.64	6.40 × 10^−2^	2.72 × 10^−2^
7	O09158	*CYP3A25*	-2.36	2.53	5.31 × 10^−4^	2.20 × 10^−4^
8	Q8K023	*AKR1C18*	1.18	-1.24	5.11 × 10^−1^	2.69 × 10^−2^
9	O35469	*HSD3B6*	1.49	-1.36	1.91 × 10^−3^	6.27 × 10^−4^
10	Q8JZK9	*HMGCS1*	6.11	-3.10	5.42 × 10^−4^	2.23 × 10^−4^
11	Q920E5	*FDPS*	2.27	-1.57	1.94 × 10^−3^	4.49 × 10^−2^
12	Q99JF5	*MVD*	3.16	-2.95	1.91 × 10^−3^	1.60 × 10^−3^
13	Q9D1G2	*PMVK*	3.33	-3.45	2.64 × 10^−3^	4.12 × 10^−3^

Note: Control group (Con). Hypercholesterolemia model group (HC). Gypenosides treatment group (GP). “-” indicates downregulation.

## Data Availability

The authors confirm that the data supporting the findings and conclusions of this study are available in the article.

## Abbreviations

The following abbreviations are used in this manuscript:

GP	Gypenosides
HFD	High-fat diet
HC	Hypercholesterolemia
TC	Total cholesterol
LDL-C	Low-density lipoprotein cholesterol
HDL	High-density lipoprotein
HSDs	Hydroxysteroid dehydrogenases
SIM	Simvastatin
